# Germline mutation rate predicts cancer mortality across 37 vertebrate species

**DOI:** 10.1093/emph/eoae016

**Published:** 2024-08-19

**Authors:** Stefania E Kapsetaki, Zachary T Compton, Walker Mellon, Orsolya Vincze, Mathieu Giraudeau, Tara M Harrison, Lisa M Abegglen, Amy M Boddy, Carlo C Maley, Joshua D Schiffman

**Affiliations:** Arizona Cancer Evolution Center, Arizona State University, Tempe, AZ, USA; Center for Biocomputing, Security and Society, Biodesign Institute, Arizona State University, Tempe, AZ, USA; Department of Biology, School of Arts and Sciences, Tufts University, Medford, MA, USA; Arizona Cancer Evolution Center, Arizona State University, Tempe, AZ, USA; University of Arizona Cancer Center, Tucson, AZ, USA; University of Arizona College of Medicine, Tucson, AZ, USA; Arizona Cancer Evolution Center, Arizona State University, Tempe, AZ, USA; Evolutionary Ecology Group, Hungarian Department of Biology and Ecology, Babeș-Bolyai University, Cluj-Napoca, Romania; Institute of Aquatic Ecology, Centre for Ecological Research, Debrecen, Hungary; Littoral Environnement Et Sociétés (LIENSs), UMR7266, CNRS Université de La Rochelle, 2 rue Olympe de Gouges, 17042 La Rochelle Cedex, France; Arizona Cancer Evolution Center, Arizona State University, Tempe, AZ, USA; Department of Clinical Sciences, North Carolina State University, Raleigh, NC 27607, USA; Exotic Species Cancer Research Alliance, North Carolina State University, Raleigh, NC 27607, USA; Arizona Cancer Evolution Center, Arizona State University, Tempe, AZ, USA; Exotic Species Cancer Research Alliance, North Carolina State University, Raleigh, NC 27607, USA; Department of Pediatrics and Huntsman Cancer Institute, University of Utah, Salt Lake City, UT, USA; Peel Therapeutics, Inc., Salt Lake City, UT, USA; Arizona Cancer Evolution Center, Arizona State University, Tempe, AZ, USA; Exotic Species Cancer Research Alliance, North Carolina State University, Raleigh, NC 27607, USA; Department of Anthropology, University of California Santa Barbara, Santa Barbara, CA, USA; Arizona Cancer Evolution Center, Arizona State University, Tempe, AZ, USA; Center for Biocomputing, Security and Society, Biodesign Institute, Arizona State University, Tempe, AZ, USA; School of Life Sciences, Arizona State University, Tempe, AZ, USA; Arizona Cancer Evolution Center, Arizona State University, Tempe, AZ, USA; Department of Pediatrics and Huntsman Cancer Institute, University of Utah, Salt Lake City, UT, USA; Peel Therapeutics, Inc., Salt Lake City, UT, USA

**Keywords:** malignancy, tumor, animals, germ cell mutations

## Abstract

**Background and objectives:**

Cancer develops across nearly every species. However, cancer occurs at unexpected and widely different rates throughout the animal kingdom. The reason for this variation in cancer susceptibility remains an area of intense investigation. Cancer evolves in part through the accumulation of mutations, and therefore, we hypothesized that germline mutation rates would be associated with cancer prevalence and mortality across species.

**Methodology:**

We collected previously published data on germline mutation rate and cancer mortality data for 37 vertebrate species.

**Results:**

Germline mutation rate was positively correlated with cancer mortality (P-value = 0.0008; R2 = 0.13). Controlling for species’ average parental age, maximum longevity, adult body mass or domestication did not improve the model fit (the change (Δ) in Akaike Information Criterion (AIC) was less than 2). However, this model fit was better than a model controlling for species trophic level (ΔAIC > 2).

**Conclusions and implications:**

The increased death rate from cancer in animals with increased germline mutation rates may suggest underlying hereditary cancer predisposition syndromes similar to those diagnosed in human patients. Species with higher germline mutation rates may benefit from close monitoring for tumors due to increased genetic risk for cancer development. Early diagnoses of cancer in these species may increase their chances of overall survival, especially for threatened and endangered species.

## BACKGROUND AND OBJECTIVES

Evolutionary forces shape germline mutation rates and cancer mortality across species. Specifically, the recent clocklike drift-barrier hypothesis [1], built upon the drift-barrier hypothesis [2, 3], states that as generation time and lifespan increase, more mutations arise, increasing the effect of selection and decreasing the effect of drift on the yearly rate of somatic and germline mutations. Then, as the effective population size decreases, the effect of drift on both somatic and germline mutation rates increases. These evolutionary forces may also affect the allele frequency of genes that protect organisms from developing cancer or, alternatively, could introduce deleterious genetic variation that increases tumor risk. The uncontrolled cell division of cancer can evolve *de novo* or can arise in the background of an inherited allele that predisposes to developing a cancer [4, 5]. This genetic risk for cancer has been observed in both humans and non-human animals [6–10]. In fact, hereditary cancer syndromes can be found in at least 10% of all pediatric and adult cancers and greatly increase the chances of tumor development [11–15]. For example, having a single BRCA1 or BRCA2 mutation in the germline increases the risk of breast cancer development in women to 60–80% [4]. Other germline variants, such as a mutant mismatch repair allele, increase the somatic mutation rate, which then leads to a dramatic increase in the risk of developing colorectal cancer [5]. Although many non-hereditary cancers can be prevented by changes in an individual’s lifestyle, hereditary cancers are harder to prevent due to the ‘first hit’ of cancer development in all their cells. Specifically, the first genetic hit refers to a mutation that occurs on one allele and inactivates the product of that gene [16]. Germ cells are haploid, and thus, if a mutation occurs in a tumor-associated recessive gene of a sperm cell and an oocyte, the zygote will have the mutation in a homozygous state. Whereas if a mutation occurs in one allele of a tumor-associated recessive gene in a somatic cell, the somatic cell has the second allele without the mutation that can mitigate the effect of the mutation in the other allele.

It is unknown whether the process of mutagenesis is similar in somatic and germline cells across the examined vertebrate species. However, there are several studies trying to better understand any possible associations between mutagenesis in germline and somatic cells, especially within humans and mice. Within humans and mice, the germline mutation rate is lower than the somatic mutation rate [17]. In humans, it has been suggested that this lower mutation rate in germline cells may be not only due to the lower cell division rate of germline cells versus somatic cells but also due to other processes, such as transcriptional scanning that repairs DNA in the germline [18]. Mutagens, such as the transposable elements L1/LINE, and different protein-truncating alleles found in several genes of human germline cells have been associated with many indels in somatic cells [19]. Liu *et al*. found a strong association between polygenic risk scores, based on germline gene variants, and the total number of somatic mutations in cancer cells [20]. Furthermore, a poor ability to prevent mutations in the germline may be associated with a poor ability to prevent secondary somatic mutations through faulty DNA synthesis fidelity or repair. Similar to increased cancer risk in humans with inherited variants in DNA repair, species with higher germline mutation rates may have higher cancer mortality compared to species with lower germline mutation rates.

Understanding the connection between inherited germline mutation rate and cancer mortality risk across different species may have a positive impact on the lives of animals through cancer screening programs and also offer new models for human genetic cancer predisposition syndromes. We focused our study on species for which data on germline mutation rates were available in public datasets. We tested whether yearly germline mutation rates across 37 vertebrate species (including 23 mammalian species, 10 bird species, 3 reptilian species and 1 species of Actinopterygii) [21] could explain variability in cancer mortality.

## METHODOLOGY

### Data collection

Germline mutation rate data were collected from previously published literature [21]. We also collected data from the literature on the domestication status of each species [8, 22, 23], their body mass [6, 24], maximum lifespan [25], average parental age [21] and trophic level (categorizing each species as either a herbivore, invertivore, primary carnivore or secondary carnivore according to its primary diet in the wild) [8, 26–30] (https://www.iucnredlist.org/).

Finally, we estimated cancer mortality risk using the Mortality and Morbidity Module of ZIMS (https://species360.org/) software for species with at least 20 records of mortality available. Only 37 out of the 68 species for which we had data on germline mutation rate had cancer mortality risk data available, with at least 20 records of mortality per species. The process of obtaining access to the raw species data centralized by Species360 has been previously described (data availability section in Vincze *et al*. [8]). To calculate cancer mortality risk for each species, we divided the total number of individuals that died of cancer by the number of individuals that died of various factors (including cancer, but excluding the number of animals that died of neonatal issues and parental neglect, as an attempt to control for infant mortality that is likely to bias cancer mortality risk estimates). We only used species with at least 20 records of mortality. Using more than 20 records per species would reduce the number of species and thus reduce the statistical power of the analyses. Using fewer than 20 records per species would add noise to the cancer mortality data. If there was no report of neoplasia, we kept the numerator as zero. In both cases of the denominator and numerator, we only used the number of animals reported as having died of a single cause of death and excluded individuals with multiple causes of death.

### Models of evolution

We used three different Markov chain Monte Carlo (MCMC) models of phenotype evolution (Ornstein–Uhlenbeck [OU], Brownian model [BM], and Early Burst [EB]) to test which model was the best fit for the germline mutation rate data across the 37 species. If the (i) OU, (ii) BM or (iii) EB model is the best fit for the germline mutation rate data, this would mean that germline mutation rates across species have mostly evolved via (i) stabilizing selection and random changes, (ii) random changes gradually accumulating or (iii) early changes in the branches of the phylogenetic tree. To test for the best fit evolutionary model, we used the fitContinuousMCMC function from the *geiger* R package [31]. The function utilizes species’ cancer mortality data and our phylogeny to fit models using maximum likelihood. This version of the function, which utilizes MCMC, gives the test the ability to incorporate informative prior distributions for node values when the information is available.

### Statistical analysis

**Figure 1.**
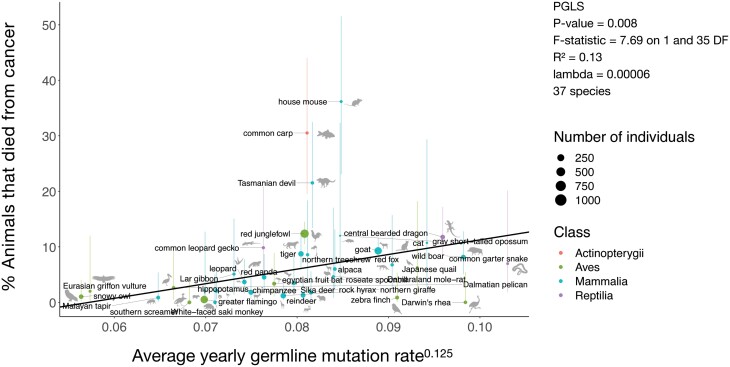
**The average yearly germline mutation rate is positively correlated with the percentage of animals (among the total number of individuals per species examined at necropsy) that died from cancer.** Each dot represents a species, and the size of the dot indicates the number of necropsies available for that species. Error bars show 95% confidence intervals. The regression line is phylogenetically controlled using the PGLS analysis. Species’ images are from PhyloPic (https://www.phylopic.org/).

We performed the phylogenetic generalized least squares (PGLS) regression analyses in R version 4.0.5 using NCBI tree creator, as described in previous studies [9, 26]. Specifically, we used a different NCBI phylogeny for each analysis, given that each analysis consisted of a different number of species. The phylogenetic tree produced by NCBI is rooted and includes branch lengths. We did not manually change branch lengths or time calibrate. We then used the R packages CAPER [32], phytools [33], geiger [34] and tidyverse [35] in the univariate analyses with cancer mortality as the dependent variable and germline mutation rate as the independent variable, and in the multivariate analysis with cancer mortality as the dependent variable and germline mutation rate and trophic level as the independent variables (see code in [36]). The germline mutation rate data did not follow a normal distribution (Shapiro’s test). By inputting the germline mutation rate data in Tukey’s test, the output provides a number (raising the values to the power of 0.125 in this case) that brings the data closer to following a normal distribution. Thus, we transformed the germline mutation rate data to the power of 0.125. We then ‘centered’ this variable by subtracting it by its mean. We also weighted the PGLS by 1/(square root of the ZIMS denominator per species) to control for the variation in animal necropsies. We added confidence intervals (95%) in the case of the cancer mortality data in Fig. 1 by using prop.test in R.

We also controlled the ‘cancer mortality ~ germline mutation rate’ analysis for the following possible confounding variables (trophic levels, average parental age, domestication, maximum longevity and body mass) due to known connections between these variables in the literature. Specifically, diet/trophic levels have been shown to significantly correlate with cancer mortality risk in mammals [8] and cancer prevalence across vertebrates [26]. Lower genetic diversity is found in carnivorous species [37]. Lifespan is significantly correlated with neoplasia prevalence (negatively) and the total number of cancers divided by the total number of neoplasias (positively) in mammals [7]. Body mass is negatively correlated with neoplasia prevalence, and the total number of cancers divided by the total number of neoplasias in mammals [7], and body mass is positively correlated with neoplasia prevalence across vertebrates [6]. Average parental age and domestication are both positively correlated with yearly germline mutation rates in mammals [21].

Next, we subsetted the dataset to only include species that had germline mutation rate, cancer mortality, adult body mass, maximum lifespan, average parental age, domestication and trophic level data. In this subset of species, we compared the AICs of univariate and bivariate models where cancer mortality was the dependent variable, germline mutation rate was always one of the independent variables, and adult body mass, maximum lifespan, average parental age, domestication or trophic level was the second independent variable. We then determined the best model fit (ΔAIC > 2).

## RESULTS AND DISCUSSION

We gathered previously published germline mutation rate data (68 species) [21] and extracted cancer mortality data (37 matching species) using the ZIMS software. By focusing only on the species that had both germline mutation rate and cancer mortality data, the common garter snake (*Thamnophis sirtalis*) had the highest germline mutation rate (1.27 × 10^−8^), and the house mouse (*Mus musculus*) had the highest cancer mortality (0.36). The snowy owl (*Bubo scandiacus*) had the lowest germline mutation rate (1.02 × 10^−10^), and the southern screamer (*Chauna torquata*), the White-faced saki monkey (*Pithecia pithecia*) and Darwin’s rhea (*Rhea pennata*) had the lowest cancer mortality (no reported cancer case).

We found that germline mutation rate was positively correlated with cancer mortality across the 37 species of our study (Fig. 1; PGLS: *P*-value: 0.0008, *F*-statistic = 7.69 on 1 and 35 degrees of freedom (DF); *R*² = 0.13; λ = 0.00006).

There were three species with relatively high cancer mortality (Fig. 1). The house mouse (36.1%: 17 animals died of cancer out of 47 individuals), common carp (30%: 18 animals died of cancer out of 59 individuals) and Tasmanian devil (21.5%: 17 animals died of cancer out of 79 individuals). Previous studies found similar high cancer prevalence in these three species. Andervont and Dunn [38] report a high prevalence of neoplasms in the house mouse (*Mus musculus*) (43.5%: 98 mice with neoplasia out of 225 necropsies). In a study across 178 species of fish, Ferraro *et al*. [39] found that the koi variety of the common carp had the highest neoplasia prevalence (18.5% neoplasia prevalence). In Boddy *et al*.’s [40] study across 37 mammalian species, Tasmanian devils were among the four species with the highest neoplasia prevalence (44% neoplasia prevalence in the Tasmanian devil species: 8 individuals with neoplasia out of 18 necropsies). We obtained cancer mortality data from Species360 which does not provide details about the reason behind neoplasm development in these animals. Future studies should identify the causation behind these relatively high cancer mortalities in these three species.

In birds and mammals, but not reptiles or fish, males have, on average, a higher germline mutation rate than females [21]. Thus, the high germline mutation rate and cancer mortality in some species could be due to the older age of their fathers [41] and/or due to diet [6]/trophic level, domestication, longer species lifespan and larger body mass. Previous analyses have identified trophic level [26], body size and gestation length (but not average adult lifespan) [6] as partial explanations for the variation in cancer prevalence across species, yet these factors only explain 1–31% of that variation. Our finding that the germline mutation rate explains 13% of the variation in cancer mortality across vertebrates (Fig. 1) suggests that inherited genetic risk should be included in future efforts to understand cancer susceptibility across species. Average parental age and domestication have been previously shown to correlate positively with germline mutation rate [21]. Although some relationships (positive, negative or none) between these variables are known [21, 42, 43], the exact associations between all variables still remain to be addressed. To account for the potential effect of these variables on cancer mortality risk, we ran bivariate regression analyses and found the following. The regression between cancer mortality and germline mutation rate is significant in bivariate analyses controlling for trophic level (37 species; *P*-value = 0.01; *F*-statistic = 6.81 on 1 and 32 DF; λ = 0.00006; *R*² = 0.19), average paternal age (months) (30 species; *P*-value = 0.02; *F*-statistic = 5.30 on 1 and 27 DF; λ = 0.99; *R*² = 0.34), domestication (37 species; *P*-value = 0.003; *F*-statistic = 9.87 on 1 and 34 DF; λ = 0.99; *R*² = 0.25), maximum longevity (months) (34 species; *P*-value = 0.006; *F*-statistic = 8.55 on 1 and 31 DF; λ = 0.00006; *R*² = 0.19) or adult body mass (grams) (34 species; *P*-value = 0.006; *F*-statistic = 8.49 on 1 and 31 DF; λ = 0.00006; *R*² = 0.18). We then compared the AICs of the univariate model of cancer mortality and germline mutation rate versus the above bivariate models. We found that the best fit model, among 28 species for which germline mutation rate, cancer mortality, average paternal age, domestication, trophic level, maximum longevity and body mass data were available, is the univariate model of cancer mortality and germline mutation rate (ΔAIC > 2). Controlling for average paternal age, domestication, maximum longevity or adult body mass did not significantly change the model fit (ΔAIC < 2). Controlling for trophic levels did not significantly improve the model fit (ΔAIC > 2).

**Figure 2.**
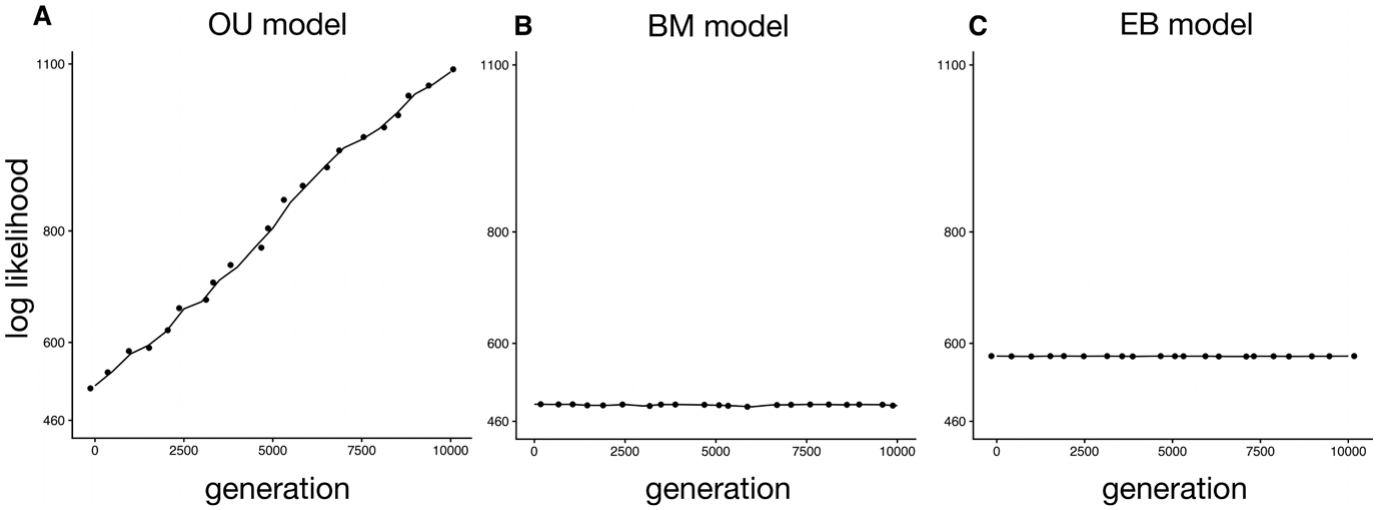
**The OU evolutionary model (A), rather than the BM (B) or EB model (C), best fits the species germline mutation rate data.** Each point represents the reported log-likelihood at each generation of the MCMC fitting algorithm. The OU model indicates that the germline mutation rate is evolving towards an optimal value, and species evolved independently. The MCMC algorithm within the fitContinuousMCMC function generates samples of the parameter estimates from the posterior distribution. The likelihood is estimated at each generation of the algorithm to determine how well the parameter estimates fit the phylogenetic tree and the germline mutation rate data.

Cancer often appears after reproductive age in humans and other species, which suggests strong selective forces in cancer defense mechanisms until later ages. Previous studies across vertebrates have found that cancer mortality risk is a trait under selection [6]. Because cancer is often lethal and can occur in animals that still have reproductive potential, we predict that the traits related to cancer suppression likely evolved under natural selection as opposed to pure random genetic drift. We analyzed whether selection or genetic drift best explains patterns of germline mutation rate across the phylogeny of our 37 species. We found that the OU model of selection best fits our data (Fig. 2, AIC: OU = −1597.09; BM = −972.47; EB = −1148.26), showing that germline mutation rate is also a trait under selection across the examined species.

The exact biological link between germline mutation rate and cancer mortality across vertebrates is unknown. There are many cross-species associations between germline mutations and the occurrence of hereditary cancers in humans and dogs, such as mutations in *BRCA1/BRCA2* and *TP53* [44]. Still, the causal relationship between germline mutation rate and cancer prevalence is still relatively unexplored across vertebrates, i.e. whether an increased germline mutation rate is associated with an increased somatic mutation rate as seen in human cancers with inherited biallelic mismatch repair deficiency [45] and/or whether increased germline mutation rate is associated with a larger burden of variants of which some may occur randomly in cancer-risk alleles leading to increased cancer rates. Reproducing our results on an even larger number of species, with additional comparative oncology databases, and understanding the interactions between life-history variables selecting for changes in germline mutation rate and cancer mortality would help to answer our central question: what explains variation in cancer susceptibility across species? For now, the species we identified with an association between germline mutation rate and increased cancer mortality may harbor species-wide hereditary risk for cancer. Similar to human patients with hereditary cancer syndromes, species with increased germline mutation rates may benefit from cancer screening to diagnose tumors at an earlier and more treatable clinical stage of development; screening that could also be performed in the future in regularly monitored endangered animals in the wild.

## Data Availability

The data and code used in this article are available in Zenodo [36].
